# Motor regulation problems and pain in adults diagnosed with ADHD

**DOI:** 10.1186/1744-9081-9-18

**Published:** 2013-05-03

**Authors:** Liv Larsen Stray, Øistein Kristensen, Martha Lomeland, Mette Skorstad, Torstein Stray, Finn Egil Tønnessen

**Affiliations:** 1 Addiction Unit, Sørlandet Hospital, Kristiansand, Norway; 2 Department of Child and Adolescent Health, Sørlandet Hospital, Kristiansand, Norway; 3 The Reading Centre, Faculty of Education and Arts, University of Stavanger, Stavanger, Norway

**Keywords:** ADHD, Motor problems, MFNU, Muscular regulation, Tonus, Inhibition, Pain, Adult

## Abstract

**Background:**

Most children who are diagnosed with attention deficit-hyperactivity disorder (ADHD) have moderate-to-severe motor problems using the Motor Function Neurological Assessment battery (MFNU). The MFNU focuses on specific muscle adjustment problems associated with ADHD, especially motor inhibition problems and high muscle tone. Here we investigated whether adults with ADHD/hyperkinetic disorder (HKD) have similar motor problems. In our clinical experience, adults with ADHD often complain about back, shoulder, hip, and leg pain. We also investigate reported pain in adults with ADHD.

**Methods:**

Twenty-five adult outpatients diagnosed with ADHD/HKD who were responders to methylphenidate (MPH) were compared to 23 non-ADHD controls on 16 MFNU subtests and using a ‘total score’ (‘TS’) parameter. The MFNU test leader was blinded to group identity. The two groups were also compared using the Pain Drawing and Numerical Pain Rating Scale.

**Results:**

The adult ADHD group had significantly (p < .001) more motor problems (higher TS) than controls. On the muscle regulation subtests, 36–96% of the ADHD group showed ‘moderate’ to ‘severe’ problems compared to 13–52% of the control group, and 80% of the ADHD group reported widespread pain. Highly significant differences were found between the ADHD and control groups for the variables ‘pain level’ (p < .001) and ‘pain location’ (p < .001). Significant correlations were found between TS and ‘pain location’ and between TS and ‘pain level’.

**Conclusions:**

These findings suggest that similar to children with ADHD, adults diagnosed with ADHD also have motor inhibition problems and heightened muscle tone. The presence of significantly higher pain levels and more widespread pain in the ADHD group compared to non-ADHD controls might indicate that pain is a long-term secondary effect of heightened muscle tone and restricted movement that can be demonstrated in children and adults by the MFNU battery.

## Background

### ADHD in Adults

Prevalence of ADHD among children and adolescents is estimated to 5,3% worldwide [1-5]. Sixty per cent of children diagnosed with ADHD in early childhood continue to demonstrate ADHD symptoms as adults [6]. Although some have argued that the severity of ADHD diminishes with age [7], others assert that the symptoms of ADHD simply change over time [8]. Specifically, symptoms such as hyperactivity are thought to diminish from childhood/adolescence to adulthood, whereas symptoms of inattention persist or even worsen with age [8]. In the context of the WHO World Mental Health Survey Initiative, researchers screened more than 11,000 people aged 18 to 44 years old in ten countries in the Americas, Europe, and the Middle East [1]. The data suggested that an average of 3.5% (range 1.2–7.3%) met the ADHD criteria. A significantly lower prevalence of ADHD was shown in low-income countries (1.9%) compared to high-income countries (4.2%). The researchers concluded that adult ADHD often co-occurs with other disorders and that it is associated with substantial impairment in adult role functioning. Although they found that few adults are treated for ADHD itself, in many instances treatment is given for co-occurring disorders. For example, up to 80% of adults with ADHD have some form of psychiatric comorbidity [9]. Less is known about somatic problems associated with the condition.

### ADHD and motor problems as assessed with the MFNU

Using the Motor Function Neurological Assessment (MFNU) [10] moderate to severe motor problems were found on all 16 subtests in 80–96% of boys with ADHD-combined [11]. Motor problems in children with ADHD are often diagnosed as the DSM-IV diagnosis Developmental Coordination Disorder (DCD) [12]. They are seen in 40-60% of these children, and typically interpreted as comorbid to the ADHD condition [13-17]. The MFNU was developed because clinical observed motor problems were not detected by standard motor test batteries like the Movement ABC-test [18] nor by the Halstead-Reitan neuropsychological test battery [10,19]. In our prior research, the MFNU revealed marked heightened muscle tone in gross movement muscles like the latissimus dorsi, sacrospinalis, iliopsoas, and calf muscles in children with ADHD [11]. High muscle tone in these muscles, which are used in a compensatory manner for stabilizing the torso, may restrict the movement of the shoulders, hips, vertebral column, and thorax. In the study by Stray et al. [11], 80% of the ADHD group and none of the control group showed highly restricted hip movement. In adolescents, lower back pain is associated with reduced hip mobility [20]. Reduced movement of the thorax may result in restriction of respiration and lead to shortness of breath [21,22]. The calf muscles (the gastrocnemius and the soleus) are active in maintaining and adjusting body alignment [23,24]. High tone in these muscles may reduce the flexibility of the foot and have a negative affect on balance. Balance problems are well known in ADHD [25-27].

Disinhibition in children with ADHD is present not only in higher order executive functions like motor planning, timing, and evaluation, but also seems to be involved in more basic motor functions. Stray et al. [10] demonstrated that children with ADHD typically exhibit a gradual increase in muscle tone when flexion-extension movements are repeated several times in succession, as in several of the MFNU subtests. This pattern, which is not seen in normal control subjects, results in a restricted movement range and in jerkiness [see videofilms in the DVD following the manual and the electronic Paper 10, 11]. These findings may explain why children with ADHD often appear clumsy and uncoordinated in daily activities [10,11], even though some of them are described by parents and teachers as being very skilled athletes. In another study, Stray et al. [28] demonstrated that a single dose of methylphenidate (MPH, Ritalin©) improved the motor inhibition problems and muscle tone in boys with ADHD and had a corresponding weaning effect after the MPH was metabolized, very similar to what is seen in the behavioural symptoms of ADHD [see videofilms 10, 28]. In a retrospective study of 73 children with ADHD symptoms (62 boys and 11 girls aged 5–17 years) the motor problems identified by the MFNU were present more often as part of the core behavioural problems of ADHD in MPH-responders than in non-responders [29]. No significant gender or age differences were found in either of the groups on any of the MFNU subtests used in that study.

These results challenge the current belief that motor problems in ADHD are either side effects or symptoms of inattention [the differential section for DCD 12, [30] or a separate (comorbid) condition [31]. The close link between positive behavioural and motor responses to MPH suggests that further research is needed to investigate the role of muscular regulation as an integrative aspect of ADHD and possibly as a physical marker of the condition. In our clinical work, we have observed that adults diagnosed with ADHD display similar patterns of motor inhibition problems and heightened muscle tone as children with ADHD [32]. Despite the high prevalence of motor problems in children with ADHD [33-37], there has been little research into whether adults with ADHD have similar problems [38].

### ADHD and musculoskeletal pain

In our clinical practice, adult patients with ADHD often report skeletal muscle pain and physical discomfort [39]. As a permanent condition, the muscular state that is associated with ADHD may elicit muscular pain and other secondary somatic effects, such as fatigue and restricted movement and respiration [40]. Kessler et al. [41] found that workers with ADHD reported significantly more chronic pain than other workers. Young and Redmond [42] found that a number of adult patients with core symptoms of ADHD also reported unexplained fatigue, widespread musculoskeletal pain, or a pre-existing diagnosis of fibromyalgia or chronic fatigue; in addition, some patients reported amelioration of pain and fatigue symptoms when medicated with ADHD medicine. In a Norwegian study, 22% of patients who were diagnosed with ADHD as adults had a history of substance abuse disease (SUD) [43]. Clinically, some reported that the SUD started after discomfort and pain that was often attributed to the negative effects of a particular lifestyle or career. One might ask if some of this discomfort and pain was also attributable to side effects of chronic muscular dysregulation that is associated with the ADHD condition itself. Little research has been done in this area.

### Aims of the present study

The aims of the present study were to investigate whether adults with ADHD exhibit the motor function problems demonstrated in children with ADHD and to investigate whether such motor problems were related to the presence of reported pain.

Our research questions and hypotheses were as follows:

1. What are the differences in motor problems in adults with ADHD as compared to non-ADHD controls as measured by the MFNU?

2. Do adults with ADHD report a) more widespread pain or b) higher levels of pain than non-ADHD controls?

We hypothesized that adults with ADHD would display consistently higher scores and show significantly more motor problems on all of the MNFU subtests used in this study as compared to non-ADHD controls. We also hypothesized that adults with ADHD would report more widespread pain and higher levels of pain than adults in the non-ADHD control group.

## Methods

### Sample

Verbal and written information about the project was given to outpatients at the Addiction Unit, Sørlandet Hospital, who were diagnosed with HKD F90.0/ADHD, had no active substance abuse, and who were positive responders to MPH. Affirmative written replies were received from 28 patients. Three of these were excluded, one for medical reasons, one who could not be taken off MPH medication due to work, and one for substance abuse. Thus the ADHD group consisted of 25 subjects with a mean age of 33 years (SD 8.9), range 20–51 years. The group included 14 men with a mean age of 34 years (SD 9.5), range 20–51 years, and 11 women with a mean age of 32 years (SD 8.3), range 20–47 years. Three subjects were university students, 12 had regular work, and 10 received social security. The patients medicated with MPH were taken off medication a least one day before the motor assessment.

The non-ADHD controls group consisted of students from the University of Agder, employees from a rehabilitation department at Sørlandet Hospital HF, and employees from a care home for the intellectually challenged in Kristiansand. Non-ADHD controls were recruited by posting flyers on bulletin boards and by study personnel and student leaders through verbal communication and written information about the project. Written affirmative answers were received from 29 subjects. To exclude possible ADHD problems in the control group, the participants were rated using the ‘M.I.N.I. Plus’ module W interview [44]. People with rheumatic diseases or with physical or medical conditions precluding participation in all of the 16 subtests of the MFNU were excluded. No precautions were taken to exclude other clinical groups. The final non-ADHD control group consisted of 23 adults without ADHD with a mean age of 41 years (SD 14.1), range 24–64 years. This group included 8 men with a mean age of 44 years (SD 14.1), range 26–64 years, and 15 women with a mean age of 40 years (SD 14.2), range 24–61 years.

### Instruments

#### MFNU

The current version of the MFNU consists of 16 subtests that are described briefly in Table 1. The tester continually monitors and guides the participant through each subtest. There is a detailed video presentation of all subtests in the DVD accompanying the Norwegian MFNU manual [10]. In an earlier study an Intraclass Correlation (ICC) of consistency, using the two-way mixed Cronbach’s model, was calculated to measure rater agreement. An average ICC of .99 (95% confidence interval, 0.98-1.00) was found, p < .001[21].

**Table 1:** The MFNU subtests used in this study [for videos see 25]

**Name of subtest**	**Description**
01. Dynamic balance-2 legs	Three sideway jumps within marked squares (back and forth). The entire process is repeated three times without stopping.
02. Dynamic balance-1 leg	Three sideway jumps on one leg within marked squares (back and forth). The entire process is repeated three times without stopping. Both legs are tested.
03. Diadochokinesis-right	Pronation-supination of one hand with the elbow flexed 90 degrees. The hand is held as an “extension” of the lower arm. The exercise is performed for approximately 15–20 seconds.
04. Diadochokinesis-left	
05. Reciprocal coordination	Alternate clenching of one fist while stretching the other in a rhythmic manner for about 15 seconds. Fingers should be almost completely extended after the hand has been clenched. Elbows are at a 90-degree angle with palms facing upwards.
06. Thumb movement	The tips of the fingers other than the thumb are successively touched with the palmar surface of the tip of the thumb. After each opposition, the subject extends and abducts the thumb. Both hands are tested for approximately 20 seconds.
07. Walking	Walking with toes alternately pointing outwards (“Chaplin”) and inwards followed by walking on the outer foot rend (Fog’s test) and inner foot rend.
08. Lifting arm	Lies prone with arms at a 45 degree angle from midline; lifts one arm with the palm of the hand facing the floor.
09. Lifting leg	Lies prone with the anterior superior iliac spine touching the floor while lifting one stretched leg at a time.
10. “Flying”	Lies prone, the arm in a 45 degree angle from midline, lifting head, arms and legs.
11. Palpation	Lies prone. The test leader palpates the back, especially the longissimus and latissimus dorsi. The test leader assesses the mobility of the thorax.
12. Passive abduction-right hip	Lies supine. Tester holds the subject’s knee and hip in a flexed position.
13. Passive abduction-left hip	The tester stretches and flexes the leg to elicit relaxation of the hip muscles and abducts the leg. The sides are evaluated separately.
14. Passive movement-right foot	Lies supine. Tester examines passive movement with dorsal flexion and eversion/plantar flexion of the right and the left feet.
15. Passive movement-left foot	
16. Synkinesis	‘Synkinesis’ is not a separate test but is an item for evaluating the synkinetic movements registered in one or more subtests. When synkinesis is observed, the tester tries to correct it. The synkinesis remaining after correction is scored.

All 16 subtests were scored by the tester or by an independent observer according to three scoring categories as described in Table 2. The scoring criteria for each scoring category on each subtest are described in greater detail in the MFNU manual.

**Table 2:** Scoring criteria for the MFNU subtests

	**Score:**	**Criteria**			
		**Subtests 01–10**	**Subtests 12–15**	**Subtest 16**	**Subtest 11**
0	No problems	The task is performed with no problems and little effort	Normal resistance against the movement is registered	Only sporadic synkinetic movements are registered	Normal muscle tone, good mobility in the thorax
1	Moderate problems	The task is performed according to instructions but requires a lot of attention and effort or performance quality is below what is expected according to age	Resistance against the movement is registered	Moderate synkinetic movements are registered in one or more subtest	Slightly greater muscle tone, some resistance against movement of the thorax
2	Severe problems	The subject cannot perform the task according to the instructions	Strong resistance against the movement is registered	Pronounced synkinetic movements are registered in one or more subtest	High muscle tone, strong resistance against movement of the thorax

Subtests 1–10 are performance tests in which the person who is being tested is instructed to perform a particular task. ‘Subtest 16: Synkinesis’ evaluates the presence of overflow movements during the MFNU assessment. Four subtests (12–15) are subtests of passive movements of the hips and feet in which the tester evaluates muscular resistance. ‘Subtest 11: Palpation’ provides important physical/proprioceptive information about muscular consistency and possible high muscle tone in muscles such as the sacrospinalis and the latissimus dorsi and about restricted movements of the thorax [10].

A reliability study of the MFNU showed high internal consistency within the total set of 17 subtests (Cronbach's alpha of .97) and a high rater agreement with an average ICC of .99 (95% confidence interval, 0.98–1.00; p < .001) [45]. Subtest 11, ‘Palpation’ was not included in this study due to problems with scoring non-visual information from the video recordings used in the evaluation procedures. The total score of the MFNU, termed the ‘TS’, can range from 0 (no problems) to 32 (severe problems, i.e. scores of 2, on all 16 subtests).

#### Pain drawing

The Pain Drawing procedure, which is commonly used in studies of pain [46-48], was chosen to categorize the patients into 4 pain groups, including a group with no pain. Areas of the body that were painful during the previous 14 days are marked on a drawing by each participant. Participants with pain localized only above a horizontal line in the thoracolumbar region (T12) were categorized into Pain group 1. Participants with pain localized only below the horizontal line were categorized into Pain group 2. Pain localized both above and below the line was defined as widespread, and subjects with widespread pain were categorized into Pain group 3. Those with no pain were categorized into Pain group 0.

#### Numerical pain rating scale

The intensity of the pain experienced during the previous two weeks was rated by each participant on an 11-point Numerical Pain Scale (NRS) that ranged from 0 (“No pain”) to 10 (“Pain as bad as it could be”) [49]. The NRS has been shown to be an appropriate measurement of pain in patients with chronic pain [50].

#### M.I.N.I. Plus

This study used the Mini-International Neuropsychiatric Interview (M.I.N.I.) version 5.0.0 with the attention deficit/hyperactivity disorder module W in the M.I.N.I.-Plus. The M.I.N.I. is an short psychiatric interview for assessing psychiatric disorders according to the DSM-IV and ICD-10 classification systems [44]. The M.I.N.I. is widely accepted and has high validity [51,52].

### Assessment

Each participant was assessed using the MFNU, Pain Drawing, and NRS. Possible ADHD symptoms in the control group were addressed by a M.I.N.I plus interview conducted by a physician or a social educator with many years of clinical and research experience in psychiatric assessment of patients with ADHD. The MNFU test leader, an experienced physiotherapist with no prior personal or professional knowledge of the subjects in either of the groups, was blinded to which group the participant was in. The assessments of the Pain Drawing, NRS, and the M.I.N.I. interview were performed prior to the MFNU assessment. The MFNU test leader did not know the results of these assessments.

### Statistical analyses

The statistical analyses were carried out using PASW (SPSS) Statistics, version 18 for Windows (SPSS Inc., Chicago, IL, USA). Descriptive statistical analyses were performed on data from each subtest in order to view the distribution of the participants (%) in the scoring categories (0, 1, or 2) and for the TS variable. As the samples and the measurements did not satisfy the conditions for parametric tests, the non-parametric Mann–Whitney U-test was used to compare the ranked scores of the ADHD group and control group on each of the 16 subtests. The Mann–Whitney U-test was used to compare the TS and the ‘Pain level’ of the ADHD group and control group for the same reason. Pearson’s Chi-square test was used to compare frequencies in the groups for the categorical variable ‘Pain location’. Spearman’s rho was run for all participants as a whole for finding the correlation between the TS of the MFNU and the ‘Pain level’ and between the TS and ‘Pain location’.

### Approval

The study was approved by The Norwegian Data Inspectorate, The National Committee for Medical Research Ethics in Norway, 2011/1947 D.

## Results

### Group differences on the MFNU

The ADHD group showed a high percentage of ‘severe problems’ (a score of 2) on many of the subtests: ‘Palpation’, ‘Passive abduction of the hip’ and ‘Passive movement of the feet’, ‘Lifting arm’, ‘Lifting leg’, ‘Diadochokinesis of the left arm’, and ‘Reciprocal coordination’. The control group typically had few problems on any of the subtests (Table 3). There were significant differences between the ADHD group and the control group for all subtests except for ‘Flying’ (Table 3).

**Table 3:** Percentages of the ADHD group (N = 25) and control group (N = 23) with the indicated motor problems on the MFNU subtests and comparison of the two groups

	**Motor problem scores of 1 or 2**					
**MFNU subtests**		**ADHD**	***Control***		**p value**	**Mann–Whitney U**
	**%**	**Median**	**%**	**Median**		
01. Dynamic balance-2 legs	56.0	1	*13.0*	0	.004	407.5
02. Dynamic balance-1 leg	76.0	2	*37.4*	0	.000	440.5
03. Diadochokinesis-right	68.0	2	*17.3*	0	.000	452.5
04. Diadochokinesis-left	80.0	2	*34.7*	0	.000	459.5
05. Reciprocal coordination	76.0	2	*17.3*	0	.000	478.5
06. Thumb movement	72.0	2	*26.1*	0	.002	422.5
07. Walking	56.0	1	*17.4*	0	.005	404.5
08. Lifting the arms	88.0	2	*39.1*	0	.000	443.0
09. Lifting the legs	84.0	2	*21.7*	0	.000	462.0
10. “Flying”	36.0	0	*13.0*	0	.102	347.5
11. Palpation	96.0	1	*52.2*	0	.000	473.5
12. Passive abduction-r. hip	84.0	2	*26.1*	1	.000	458.5
13. Passive abduction-l. hip	84.0	2	*25.7*	0	.000	464.0
14. Passive movement-r. foot	84.0	2	*17.2*	0	.000	481.0
15. Passive movement-l. foot	84.0	2	*17.4*	0	.000	471.0
16. Synkinesis	60.0	2	*26.0*	0	.027	383.5

Scoring: 0, ‘No problems’; 1, ‘Moderate problems’; 2, ‘Severe problems’.

The Mann–Whitney U-tests (Table 3) showed that the ADHD group had significantly more motor problems (higher TS; median = 25) than the control group (median = 2) (U = 485.00, p < .001). The mean TS of the ADHD group was 21.20, SD = 9.5, and the mean TS of the control group was 6.30, SD = 9.9, Cohen's d of the TS between the groups was 1.52, indicating a large effect size [53]. There were no significant gender differences in either of the groups as shown by Mann–Whitney U-test analysis of the TS. Most subjects in the control group had no problems on the MFNU. Three subjects had high TS, and all three showed both inattention and impulsive ADHD problems on the M.I.N.I. Plus, although they did not fulfil the ADHD diagnostic criteria.

### Group differences in ‘pain location’ and ‘pain level’

The Pearson’s Chi-Square test showed significant differences between the ADHD group and the control group for the variable ‘Pain location’ (P < .001). Widespread pain was reported by 80% of the ADHD group compared to 17.4% of the control group. Only 8% of the ADHD group reported having no pain in the previous two weeks compared to 34.8% of the controls. There were no significant gender differences regarding pain level or pain location in either of the groups (Table 4).

**Table 4:** Percentages of the ADHD group and control group with pain at the indicated location

**Pain location**	**Group**	
	**Control (N = 23)**	**ADHD (N = 25)**
No pain	34.8%	8.0%
Localized pain (only in upper body)	34.8%	8.0%
Localized pain (only in lower body)	13.0%	4.0%
Widespread pain	17.4%	80.0%
Total	100%	100%

The Mann–Whitney U-tests showed a significant difference in self-reported pain (‘Pain level’) between the ADHD group (mean rank = 31.76, median =5) and the control group (mean rank = 16.61, median = 1) (U = 469.00, p < .001). The mean ‘Pain level’ for the ADHD group was 4.76, SD = 2.16, and the mean ‘Pain level’ for the control group was 1.96, SD = 2.27. The Cohen’s d for ‘Pain level’ was 1.26, which is a large effect size. There was a significant correlation (p < .01) between the TS on the MFNU and ‘Pain level’ and between the TS and ‘Pain location’ (p < .05) (Spearman's rho).

## Discussion

### Motor problems

We hypothesised that adults with ADHD would have more problems than controls in regulating movement and muscle tone as assessed by the MFNU. This hypothesis was supported by the results on all subtests except the ‘Flying’ subtest. These results strongly suggest that muscle regulation problems are very similar in adults with ADHD as in children with ADHD. Most of the ADHD subjects had a moderate to severe TS, indicating that just as in children, the adults had motor problems that affected many facets of movement and muscle control. Comparison of the results for the adult ADHD group to results for 8–12-year-old boys showed minor differences in two subtests. The results on subtest 16, ‘Synkinesis’, and subtest 07, ‘Walking’ indicated that adults with ADHD may be less affected by overflow movement (synkinesis) than children (see [11]). Overflow movements reflect immaturity of the cortical systems involved in automatic inhibition [54] and are also present in many children without ADHD. Overflow movements are normally less apparent in adolescence and adulthood than in childhood, which is the natural consequence of more mature nervous systems [55,56]. Thus, it is likely that the low scores on the ‘Synkinesis’ subtest in adults with ADHD compared to children with ADHD are due to maturation. Even so, our results indicated that adults with ADHD have more problems with overflow movement than non-ADHD adults. Similarly, for subtest 10, ‘Flying’, more subjects in the ADHD group had problems compared to the control group, but a high percentage of the ADHD group showed normal performance on this subtest.

Motor problems in ADHD have been seen as being co-effects or a motor consequence of inattention and impulsivity [12]. This view has been challenged by several researchers. In fact, some of these have noted that the poor fine motor abilities of children with ADHD cannot be attributed to deficits in attention or concentration but rather are due to factors related to motor ability [14,15]. ADHD and motor problems may have a common basis that may be due to genetic factors and/or shared environmental factors [57]. Few of the subtests of the MFNU require focused attention for more than half a minute, and the subject is continually monitored and guided throughout each subtest, thus preventing distraction. As in children with ADHD [11,29], motor inhibition problems were highly present in the adult ADHD group as demonstrated by subtests 03 and 04, ‘Diadochokinesis’, subtest 06, ‘Thumb movement’, and subtest 05, ‘Reciprocal inhibition’. Similar to findings in children in our previous study [11], a very high percentage of the adult ADHD group showed problems on subtests that reveal heightened muscle tone in the latissimus dorsi, iliopsoas, and the gastrocnemius-soleus (calf) muscles (subtest 08, ‘Lifting arm’, subtests 12 and 13, ‘Passive abduction of the hips’, subtest 09, ‘Lifting leg’, and subtests 14 and 15, ‘Passive movement of the feet’). Heightened muscle tone was also demonstrated in the sacrospinalis by subtest 11, ‘Palpation’. None of these subtests are likely to be influenced by inattention or impulsivity since they are either closely monitored by the tester or are performed by the tester.

It is often argued that motor problems in ADHD that are not explained by inattention are comorbid symptoms of Developmental Coordination Disorder (DCD) [30,58], which is a motor skills disorder. While many people with ADHD may have a DCD condition, as measured by standardised motor test batteries, the motor and muscular patterns assessed by the MFNU are not necessarily associated with DCD. Many years of clinical experience using the MFNU has shown that people with ADHD may demonstrate above-average motor skills and be skilful athletes but still have a high MFNU problem score. Our findings in children, which are corroborated by the present study in adults with ADHD, suggest that the motor problems revealed by the MFNU are different in nature from deficits in motor skills (see [34]). Stray [21] argues that the motor disinhibition and heightened muscle tone of people with ADHD may be directly related to neurofunctional processes associated with dysregulation of the dopamine system and possibly also the noradrenaline system, which are currently thought to be central to the ADHD condition [59].

Recent research has focused on the frontostriatal system and basal ganglia [60,61]. Not only the mesocortical and mesolimbic systems, but also the nigrostriatal pathways [62], the cerebellum [63] and the reticular formation [64-66] are affected in ADHD. These pathways play important roles in regulating fine motor control and in whole body stabilisation. It is well known that reticular formation is involved in regulation of arousal [67], which is thought by many to be important for understanding the ADHD condition [68-70]. In this context, it is interesting that the reticulospinal systems are important in the regulation of postural control [71], in the regulation of movements [72,73], and in reducing muscle activity in rapid-eye-movement (REM) sleep [73]. The reticular system has an important regulatory role in the maintenance of muscle tone based on the balanced influence of the inhibitory and facilitating regions of the reticular formation [74]. Neurons from the reticular formation can activate a whole set of muscles at the same time, possibly modulating adjustments in body position that are needed to maintain balance and affecting the stabilizing muscles (the proximal extremity muscles and muscles that stabilize the vertebral column) [75]. Our findings might suggest a functional link between these two functions of the reticular system, i.e. maintenance of muscle tone and stabilization of motor control, and the regulation of arousal. The findings might further suggest that both processes are concurrently influenced by the central stimulating effect of MPH. It is still unclear, however, why people with ADHD would have higher muscle tone than normal and why MPH helps normalize muscle tone [28]. Problems with motor inhibition in ADHD are well known [76,77]. As mentioned, many of these problems may be bodily expressions of inattention and impulsivity rather than specific motor problems. However, many of the MFNU subtests reflect motor inhibition problems that seem to be related to dysregulation of the release of, or inhibition of, active muscles. This is demonstrated by tasks that involve rapid repetition of a simple movement (e.g. ‘Thumb movement’ and ‘Diadochokinesis’). Most often, people with ADHD have no initial problems performing such tasks. However, the movements typically become increasingly jerky and uncoordinated with repetition and involve great effort. MPH has an immediate effect on these release problems (see videos in [10,28]), and there is a gradual decrease of the effect as MPH is metabolized [28]. This further suggests a functional connection between the demonstrated problems in regulating muscle activity and dysregulation of higher executive functions thought to be involved in ADHD. In light of these findings, one might ask if muscle release problems are involved in gross movements such as walking and running as well as in fine motor skills such as handwriting. Fliers et al. [78] found an association between motor problems in ADHD and genes involved in muscles function.

### Pain

As we hypothesized, the ADHD group reported significantly more widespread pain (both in the upper and lower body) and a higher pain level than the control group. To our knowledge this has not been assessed before. The highly significant correlation between the TS and the pain level score might suggest that the pain reported in the ADHD group is directly linked to motor problem severity and that such pain might be an indirect long-term consequence of the muscle tone dysregulation and motor inhibition that is associated with the ADHD condition. A history of childhood ADHD, assessed with the DSM-IV criteria, has been shown to be more common in the patients diagnosed with Fibromyalgia compared to normal controls (32.3% vs. 2.52%) [79]. Such a relationship has to be addressed in a future study. Persistent pain may negatively affect attention [80], daily living [81], and quality of life [82]. There is a well known dilemma between the use of painkillers and abuse or addiction [83]. In the future, it would be interesting to look at the consumption of painkillers, especially opioids, among patients with adult ADHD.

## Limitations

In this study, the control group had a higher mean age (41 years) than the ADHD group (32 years). This might have influenced the results, although it seems unlikely that there is an inherent reason why older people would be expected to perform better on the MFNU or have less pain than the younger people in the ADHD group. Significant differences were found on the same subtests using age matched participants. The subjects diagnosed with ADHD who were included in this study were all responders to MPH for their ADHD symptoms. This implies that our conclusions cannot be generalized to the broader ADHD population. The individuals in the ADHD group had an earlier history of addiction problems. Even if substance abuse was controlled for in this study, the confounding effects of residual pain or secondary health problems due to prior substance abuse cannot be ruled out. Further research involving a sample of adults without prior substance abuse is needed to clarify this issue.

## Conclusions

This study demonstrated that MPH-responsive adults with ADHD show the same functional motor problems as children with ADHD as measured with the MFNU. The motor problems were associated with heightened muscle tone in gross movement muscles and with specific motor inhibition problems that resulted in restricted movement and instability. The MFNU was able to effectively discriminate between adults with ADHD and non-ADHD controls. The ADHD group also differed significantly from the control group in terms of reported pain in that the ADHD subjects reported more widespread pain and higher levels of pain than the controls. The ADHD group showed high correspondence between reported muscular pain and TS on the MFNU. These findings suggest that the demonstrated functional motor problems are important contributors to the very high incidence of reported pain in the ADHD group and possibly to the substance abuse and addiction problems that are common in people with ADHD. Further research validation with different methods is needed to confirm these conclusions.

## Consent

Written informed consent was obtained from the patient for publication of this report.

## Competing interests

The MFNU manual is sold by the Reading Centre, University of Stavanger, for NOK 700 (about €85). LLS receives 9% royalties and TS receives 3% royalties from the sale of the MFNU.

## Authors’ contributions

LLS was the primary contributor to the study conception and design and was mainly responsible for data acquisition, analysis, and interpretation. LLS was the main contributor to drafting of the manuscript. ØK contributed to the study conception and design, helped interpret the data, and helped draft the manuscript. ML contributed to the study conception and design and helped acquire the data. MS helped acquire the data. TS contributed to data analysis and helped draft the manuscript. FET contributed to data analysis and helped draft the manuscript. All authors read and approved the final manuscript.
